# DNA Sequence Analysis of Plasmids from Multidrug Resistant *Salmonella enterica* Serotype Heidelberg Isolates

**DOI:** 10.1371/journal.pone.0051160

**Published:** 2012-12-10

**Authors:** Jing Han, Aaron M. Lynne, Donna E. David, Hailin Tang, Joshua Xu, Rajesh Nayak, Pravin Kaldhone, Catherine M. Logue, Steven L. Foley

**Affiliations:** 1 Division of Microbiology, National Center for Toxicological Research, United States Food and Drug Administration, Jefferson, Arkansas, United States of America; 2 Department of Biological Sciences, Sam Houston State University, Huntsville, Texas, United States of America; 3 Marshfield Clinic Research Foundation, Marshfield, Wisconsin, United States of America; 4 ICF International Incorporation at NCTR/FDA, Jefferson, Arkansas, United States of America; 5 Department of Veterinary Microbiology and Preventative Medicine, Iowa State University, Ames, Iowa, United States of America; University of Birmingham, United Kingdom

## Abstract

*Salmonella enterica* serovar Heidelberg is among the most detected serovars in swine and poultry, ranks among the top five serotypes associated with human salmonellosis and is disproportionately associated with invasive infections and mortality in humans. *Salmonella* are known to carry plasmids associated with antimicrobial resistance and virulence. To identify plasmid-associated genes in multidrug resistant *S. enterica* serovar Heidelberg, antimicrobial resistance plasmids from five isolates were sequenced using the 454 LifeSciences pyrosequencing technology. Four of the isolates contained incompatibility group (Inc) A/C multidrug resistance plasmids harboring at least eight antimicrobial resistance genes. Each of these strains also carried a second resistance plasmid including two IncFIB, an IncHI2 and a plasmid lacking an identified Inc group. The fifth isolate contained an IncI1 plasmid, encoding resistance to gentamicin, streptomycin and sulfonamides. Some of the IncA/C plasmids lacked the full concert of transfer genes and yet were able to be conjugally transferred, likely due to the transfer genes carried on the companion plasmids in the strains. Several non-IncA/C resistance plasmids also carried putative virulence genes. When the sequences were compared to previously sequenced plasmids, it was found that while all plasmids demonstrated some similarity to other plasmids, they were unique, often due to differences in mobile genetic elements in the plasmids. Our study suggests that *Salmonella* Heidelberg isolates harbor plasmids that co-select for antimicrobial resistance and virulence, along with genes that can mediate the transfer of plasmids within and among other bacterial isolates. Prevalence of such plasmids can complicate efforts to control the spread of *S. enterica* serovar Heidelberg in food animal and human populations.

## Introduction

*Salmonella enterica* is a major cause of foodborne illnesses. In the United States alone, over 1 million cases of salmonellosis are predicted to occur annually [1]. The economic cost associated with these infections has been estimated to be between 2.3-billion to 9.4-billion US dollars each year due to loss of work, medical care, quality of life and death associated with salmonellosis [2]–[4]. Among the over 2,500 serovars of *S. enterica* identified, serovar Heidelberg is among the top five most commonly identified serotypes in human salmonellosis and is commonly detected among *Salmonella* isolates submitted to the National Veterinary Services Laboratory that originated from poultry and swine [5]. Annually, infections with *S.* Heidelberg lead to approximately 84,000 cases of salmonellosis and contribute to approximately 7% of the *Salmonella*-related deaths in the U.S.; the second highest percentage after *S.* Typhimurium [6], [7]. Poultry products such as turkey, chicken and eggs are important sources of *S.* Heidelberg infections in humans [7]. Contamination of poultry products with *Salmonella* remains an important concern because of consumer preferences for poultry products. Data from the U.S. Department of Agriculture (USDA) indicate nearly four-fold increase in the per capita consumption of poultry products in the U.S. over the past half century [8].

While the majority of *Salmonella* infections are relatively mild, self-limiting, and usually resolve within a few days, *S.* Heidelberg tends to cause a disproportionately high percentage of invasive infections [9]. Analysis of the FoodNet data by the Centers for Disease Control and Prevention (CDC) found that *S.* Heidelberg were responsible for 11% of invasive infections [9], but accounted for only 5.6% of all reported cases of salmonellosis during the study period [10]. These invasive infections can potentially cause severe pathology and death, thus requiring antimicrobial therapy. Consequently, development of antimicrobial resistance among *S.* Heidelberg isolates has been a concern. Currently, fluoroquinolones and extended spectrum cephalosporins are the first-line drugs for treating severe *Salmonella* infections [11]. Because fluoroquinolones are not approved for use in pediatric patients, severe *Salmonella* infections in children are treated with cephalosporins [11]. People under the age of 20 accounted for ∼46% of all *Salmonella* infections in patients with known ages reported to the CDC [5]. Unfortunately, there has been an increase in the emergence and spread of cephalosporin-resistant strains of *Salmonella* over the last decade [12]. Data from the National Antimicrobial Resistance Monitoring System (NARMS) indicated that the percentage of *S.* Heidelberg isolates from humans and poultry (chicken and turkey) that were resistant to cepholosporins has increased from 1997 to 2008. For example, in 1997, none of the isolates from humans and 1.6% of poultry were resistant to ceftiofur, by 2008 the numbers increased to 8.0% and 21%, respectively [12]. This increase in cephalosporin resistance is likely associated with the spread of the AmpC β-lactamase, which is encoded by *bla*_CMY_ [13], [14]. The *bla*_CMY_ gene has been associated with transmissible plasmids and could facilitate the spread of cephalosporin resistance [13].

Additionally many of the cephalosporin-resistant strains also display multidrug resistance (MDR) and often these MDR phenotypes can be transferred *en masse* to susceptible strains [15]–[18], which can present an additional challenge for controlling *S*. Heidelberg in food-animal production settings. Welch *et al.* (2007) described the sequence of a large transmissible incompatibility group (Inc) A/C plasmid from a *S.* Newport strain carrying 11 resistance determinants and provided compelling evidence that similar IncA/C plasmids are widely distributed in isolates of various *Salmonella* serovars, including *S.* Heidelberg [19]. In 2011, a multi-state outbreak of human *S*. Heidelberg involving 34 U.S. states was linked to the consumption of ground turkey that resulted in a total of 136 infections (http://www.cdc.gov/salmonella/heidelberg/111011/index.html). The outbreak strain of *S*. Heidelberg was resistant to multiple commonly prescribed antimicrobials, which likely increased the risk for treatment failure in the infected individuals. The spread of multidrug resistance among *S.* Heidelberg isolates can pose a threat to the management of salmonellosis in animal husbandry and human medicine. Since many of antimicrobial resistance and virulence genes are encoded on plasmids, an understanding of plasmid genetics is important for comprehending the factors associated with increased antimicrobial and virulence resistance in *S.* Heidelberg. The objective of this study was to sequence multidrug resistance plasmids isolated from *S.* Heidelberg and compare the data to previously sequenced plasmids. These results provide additional data to better understand the genetics of plasmid-associated antimicrobial resistance in *S.* Heidelberg.

## Materials and Methods

### Bacterial Strains

Five multidrug resistant *S. enterica* serovar Heidelberg isolates were selected for plasmid sequencing based on their combinations of MDR phenotypes, pulsed-field gel electrophoresis and plasmid profiles described previously [15], [16], [20]. These isolates included isolate 111 (bovine diagnostic specimen from Ohio collected in 2001), 146 (porcine diagnostic specimen from Minnesota in 2002), 163 (turkey diagnostic specimen from Ohio in 2002), 696 (turkey carcass swab from a Midwestern processing facility in 2000) and 1148 (human patient from Wisconsin received in 2008). Based on the combinations, the isolates were distinct from one another, but shared similar characteristics with other *S.* Heidelberg isolates in the previous studies [15], [16], [20]. The isolates were maintained at –80°C in freezer vials containing Brain Heart Infusion broth supplemented with 20% glycerol.

### Plasmid Isolation

Plasmid DNA was isolated by the methods described by Wang and Rossman for the isolation of both large and small sized plasmids [21]. The DNA concentrations of the plasmid extracts were calculated by measuring the absorbance at A_260_ and A_280_ using the NanoDrop ND-1000 Spectrophotometer (NanoDrop; Wilmington, DE) to ensure that there was adequate plasmid DNA for sequencing. The plasmid DNA was frozen until submission for sequencing.

### Plasmid Sequencing, Assembly and Annotation

Isolated plasmid DNA was sent to Roche 454 LifeSciences Sequencing Center, Bradford, CT or the David H. Murdoch Research Institute, Kannapolis, NC for 454 pyrosequencing and the sequencing reads were quality checked and initially assembled using the Newbler program (Roche 454 LifeSciences, Bradford, CT) [22], [23]. The initially assembled contigs were analyzed using the SeqMan Pro (Lasergene 8.0, DNAStar, Madison, WI) and the National Center for Biotechnology’s Basic Local Alignment Search Tool (BLAST) [24]. Briefly, unique ends on each of the contigs were identified using the contig extension feature of SeqMan Pro. The extended ends were compared to each of the other sequence contigs using the BLAST program to identify regions of overlap that would indicate that the contigs are attached, thus filling any gaps. The end-matching process was repeated until individual plasmids were closed. PCR primers were designed to amplify the regions where the large contigs were joined. Positive PCR products were bi-directionally sequenced from the PCR primers using the Big Dye terminator kit with an ABI 310 sequencer (Applied Biosystems, CA) to verify proper assembly. In addition, individual sequence reads were mapped back to the assembled plasmids using the Genomics Workbench (CLC bio, Aarhus, Denmark) to confirm that there were continuous overlapping reads over the entire length of the assembled plasmid.

Following completion of plasmid assembly, the plasmid sequences were submitted to the RAST annotation pipeline (Argonne National Laboratory, Argonne, IL) to identify putative coding sequences (CDS) and provide an initial annotation. These annotations were manually evaluated using Artemis (Sanger Institute, Cambridge, UK). The CDS were examined to verify, and update if needed, the gene and protein identities following comparison to GenBank sequences using BLAST [24]. CDS that did not match an entry in the GenBank database were identified as encoding hypothetical proteins. The completed plasmid sequences were visualized using the DNAplotter program (Sanger Institute) and were deposited in GenBank with accession numbers, JN983042 to JN983049.

### Comparative Genetic Analysis

To initially characterize the plasmids, incompatibility (Inc) groups were determined *in silico*, by mapping the PCR primers described by Carattoli et al. [25] to the assembled plasmid sequences with BLAST configured for short reads. Additionally, the plasmids were evaluated for the presence of known genes associated with antimicrobial and disinfectant resistance, virulence and plasmid transfer, as well as mobile genetic elements such as integrons and transposons, based on the annotations and BLAST searching. The assembled plasmid sequences were submitted to BLAST and compared to previously sequenced plasmids in GenBank as of August 15, 2012. Plasmids with highly similar sequences were identified and analyzed using the BLAST pairwise alignment to construct neighbor joining trees to identify the plasmids with the highest degree of similarity to the plasmids in the study. Additionally plasmids with greater than 50% query coverage were imported into the Mauve (ver. 2.0) program to conduct multiple alignments to compare the distribution of determinants, such as those potentially associated with antimicrobial resistance, virulence and transfer, identified in the sequenced plasmids with those in the GenBank database [26].

## Results

**Figure 1:**
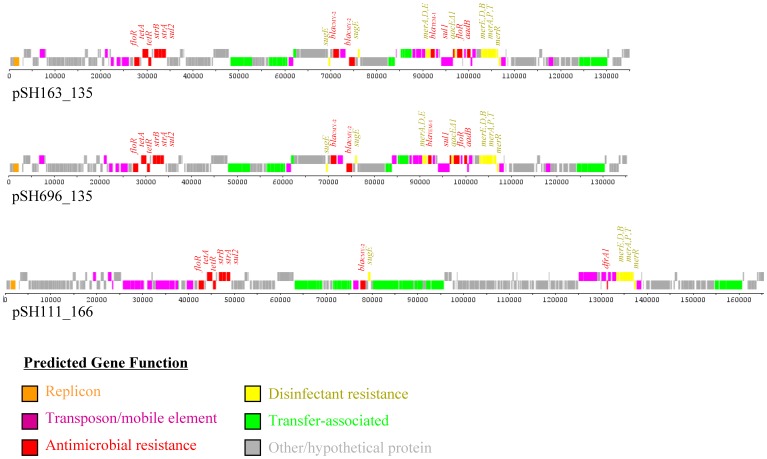
Linear representation of the IncA/C plasmids. For each of the plasmid diagrams the predicted functions of genes are identified by the colors indicated in the figure key. The predicted antimicrobial and disinfectant/heavy metal resistance genes are identified in plasmids pSH163_135, pSH696_135 and pSH111_166.

**Figure 2:**
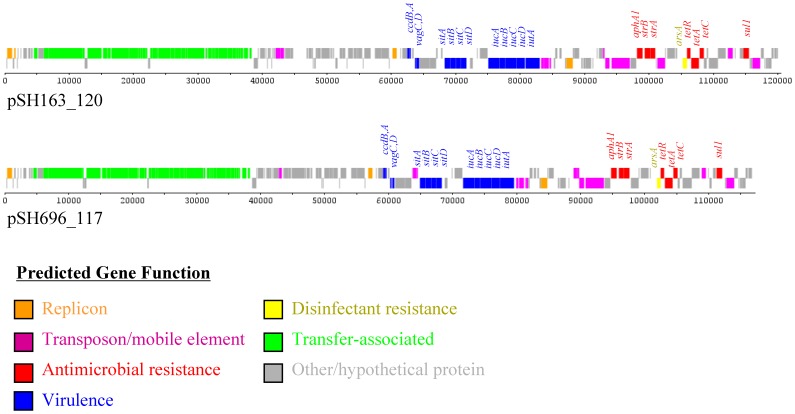
Linear representation of the IncFIB plasmids. For each of the plasmid diagrams the predicted functions of genes are identified by the colors indicated in the figure key. The predicted virulence, antimicrobial and disinfectant/heavy metal resistance genes are identified in plasmids pSH163_120 and pSH696_117.

DNA sequencing of plasmids from five multidrug resistant *S. enterica* serovar Heidelberg isolates showed that each isolate studied had at least one smaller plasmid of less than 10 kb in size (all without antimicrobial resistance genes) along with larger plasmids greater than 100 kb (Table 1). This study focused on the sequencing results and characterization of those plasmids containing antimicrobial resistance determinants. Four of the five isolates in the study harbored two plasmids containing antimicrobial resistance genes that were consistent with the observed susceptibility profiles for the isolates. With the exception of isolates 163 and 696, the plasmids from the isolates were quite different from each other. Isolate 163, which originated from a turkey diagnostic specimen, contained resistance plasmids of approximately 135 and 121 kb in size, as well as a 34 kb plasmid carrying genes associated with VirB/D4 Type IV Secretion System (T4SS; pSH163_34, accession number JX258656) and two small 3.4 and 3.3 kb plasmids that are very similar to CP001119 and CP001149, respectively. Similarly, isolate 696 which was collected from a turkey processing plant contained resistance plasmids of 135 and 117 kb and the VirB/D4 T4SS (pSH696_34, JX258654), 3.4 and 3.3 kb plasmids similar to isolate 163. The 135 kb plasmids from isolates 163 (pSH163_135; accession number JN983045) and 696 (pSH696_135; JN983048) were members of the IncA/C type and clustered most closely to one another in comparison to previously sequenced IncA/C-like plasmids (Figure 1). The plasmids share similar sets and arrangement of genes in their genetic backbone and carry the same sets of antimicrobial resistance (Table 1) and disinfectant resistance genes, including those associated with mercury and quaternary ammonium compound resistance (Figure 1). Both isolates carried an additional resistance plasmid, which are IncFIB plasmids of approximately 120 kb (isolate 163; pSH163_120; JN983046) and 117 kb (isolate 696; pSH696_117; JN983047) in size (Figure 2). Each plasmid contained genes associated with resistance to kanamycin (Kan), streptomycin (Str), sulfonamides (Sul) and tetracyclines (Tet; Table 1). These IncFIB plasmids also contained multiple iron acquisition genes, including those of the aerobactin (*iucABCD* and *iutA*) and the Sit iron transport (*sitABCD)* operons (Figure 2).

**Table 1:** Bacterial isolates used in this study and their characteristics.

Isolate	Antimicrobial Resistance Profile^A^	Plasmid(bp)	Plasmid Name	Accession Number	Inc. Group^B^	Resistance Genes Identified
111	Chl, Tet, Axo, Tio, Fox, Amp,Amc, Gen, Kan, Str, Sul, Sxt	165,791	pSH111_166	JN983043	A/C	*aacC, bla*_CMY_, *dfrA1, floR, strA, strB, sul2, tetA*
		227,608	pSH111_227	JN983042	HI2	*neoR, sph, strA, strB, tetA, tetC, tetD*
146	Chl, Tet, Axo, Tio, Fox, Amp,Amc, Kan, Str, Sul, Sxt	65,030	pSH146_65	JN983044	None	*bla*_CMY_
		>165,000^C^	pSH146_A/C	N/A	A/C^C^	C *aadA, aphA, bla*_CMY_, *dfrA12, floR, strA, sul1, sul2, tetA*
163	Chl, Tet, Tio, Fox, Amp, Amc,Gen, Kan, Str, Sul	135,168	pSH163_135	JN983045	A/C	*aadB, bla*_CMY_, *bla*_TEM_, *floR, cmlA, strA, strB, sul1, sul2, tetA*
		120,524	pSH163_120	JN983046	FIB	*aphA, strA, strB, sul2, tetA, tetC, tetD*
696	Chl, Tet, Tio, Fox, Amp, Amc,Gen, Kan, Str, Sul	135,423	pSH696_135	JN983048	A/C	*aadB, bla*_CMY_, *bla*_TEM_, *floR, cmlA, strA, strB, sul1, sul2, tetA*
		117,278	pSH696_117	JN983047	FIB	*aphA, strA, strB, sul2, tetA, tetC, tetD*
1148	Gen, Str, Sul	106,833	pSH1148_107	JN983049	I1	*aacC, aadA, sul1*

A Antimicrobial abbreviations: chloramphenicol (Chl), tetracycline (Tet), ceftriaxone (Axo), ceftiofur (Tio), cefoxitin (Fox), ampicillin (Amp), amoxicillin/clavulonic acid (Amc), kanamycin (Kan), streptomycin (Str), sulfamethoxazole (Sul) and trimethoprim/sulfamethoxzole (Sxt).

B Plasmid Incompatibility (Inc) group.

C Plasmid sequence was not able to be fully assembled due to lack of coverage (typically 2–4 fold), the size was estimated following gel electrophoresis of isolated plasmid DNA and the detection of genes present were based on the mapping of unassembled sequence reads to a series of IncA/C reference sequences (AB277723, AB571791, CP000603, CP000604, FJ621586, HQ02386, and JF14412).

**Figure 3:**
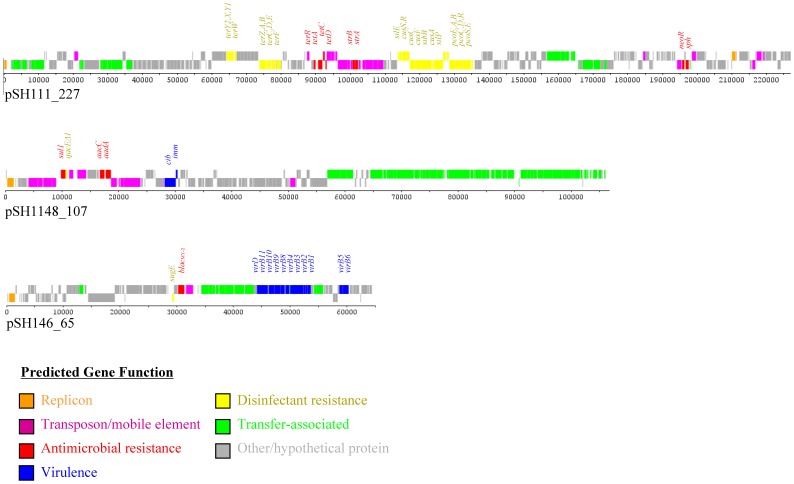
Linear representation of the other antimicrobial resistance plasmids sequenced in the study, including the IncHI2 plasmid pSH11_227, the IncI1 plasmid pSH1148_107 and pSH146_65, which was not classified by the Inc grouping. The predicted functions of genes are identified by the colors as indicated in the figure key and the predicted virulence, antimicrobial and disinfectant/heavy metal resistance genes are identified in each of the plasmids.

Isolate 111 contains two large multidrug resistance plasmids, an IncA/C plasmid that is approximately 166 kb (pSH111_166; JN983043; Figure 1) and an approximately 227 kb IncHI2 plasmid (pSH111_227; JN983042; Figure 3). The IncA/C plasmid, pSH111_166, harbored genes associated with resistance to at least eleven antimicrobials (Table 1), including trimethoprim, which is distinct from isolates 163 and 696 as the trimethoprim resistance gene (*dfrA1*) was not detected in these two isolates. The plasmid also contained genes associated with resistance to quaternary ammonium compounds and mercurial agents (Figure 1). pSH111_227 also contained multiple antimicrobial resistance genes (Table 1) along with a large number of heavy metal resistance genes, including those associated with resistance to tellurium (*terABCDEFWXYZ*), silver (*silE*), copper (*pcoABCDERS*), cobalt, zinc and cadmium (*cusACFRS*) (Figure 3).

Isolate 1148 had a single 107 kb IncI1 resistance plasmid, pSH1148_107 (JN983049), and one small 4.8 kb plasmid (JX494965). The IncI1 resistance plasmid encoded antimicrobial resistance to gentamicin (Gen), Str and Sul (Table 1) and disinfectant resistance genes that are associated with resistance to the quaternary ammonium compounds (Figure 3).

Isolate 146 had four plasmids, two of which contained antimicrobial resistance genes. One was a 65 kb plasmid (pSH146_65; JN983044) that was untypeable using the PCR primers for the replicon typing scheme. The plasmid contained the resistance gene *bla*_CMY-2_ which is associated to resistance to a number of the β-lactams, including the extended spectrum cephalosporins (Figure 3). A second resistance plasmid (>165 kb) was detected in isolate 146 based on antimicrobial susceptibility testing, conjugation and replicon typing results (data not shown). However, the sequence reads associated with this plasmid were limited (typically 2 to 4-fold coverage); thus, full assembly of this plasmid was not possible. Based on replicon typing results, the plasmid was likely an IncA/C plasmid. The reads were mapped to a diverse group of previously sequenced IncA/C to identify resistance genes likely present in the plasmid (Table 1). Sequence mapping revealed the presence of at least eight different resistance genes, including *aadA, aphA, bla*_CMY-2_, *dfrA12, floR, strA, sul1, sul2* and *tetA*, which correspond to the observed resistance phenotypes for the isolate (Table 1). Beside the two resistance plasmids, isolate 146 also contained a 32 kb VirB/D4 T4SS-containing plasmid (JX258655) and a IncI1 plasmid without additional resistance genes (pSH146_87, JX445149).

The results of the fully assembled plasmids were submitted for BLAST searching to identify cohorts of plasmids that had a high degree of sequence similarity. These plasmid sequences were analyzed using the BLAST pairwise alignment feature to construct neighbor joining trees to infer which plasmids demonstrate the highest degree of similarity to the plasmids characterized in the study. The IncA/C plasmids pSH163_135 and pSH696_135 clustered together and were most similar to a plasmid isolated from *Escherichia coli* (accession number FJ621586). The pSH111_166 were grouped in a cluster with multiple plasmids from *Salmonella* and *Escherichia coli*. The IncFIB plasmids (pSH163_120 and pSH696_117) were most similar to one another and clustered with plasmids isolated from four *E. coli*, pAPEC-O1-ColBM (DQ381420), pChi7122 APEC-1 (CP000836) pVM O1 (EU330199), and pO83_CORR (CP001856), that were isolated from three avian pathogenic *E. coli* (APEC) strains and a Crohn’s disease patient, respectively. The IncI1 plasmid pSH1148_107 clustered most closely *S.* Typhimurium plasmid TY474p2 (CP002489) and *Escherichia coli* plasmid pND11_107 (HQ11428). Plasmid pSH111_227 was closely related to plasmid pAPEC-O1-R (DQ517526), isolated from APEC isolate [27], and distant from the remaining four IncHI2 isolates. The regions in pSH111_227 that were divergent from pAPEC-O1-R were associated with transposable elements carrying Tet and Kan resistance genes (Figure 3). pSH146_65 was most similar (>70% query coverage) to plasmids R721 from *E. coli* (AP002527) and pChi7122-3 (FR851304) from an APEC strain.

## Discussion

This study highlights the sequencing results of antimicrobial resistance plasmids from five multidrug resistant *S.* Heidelberg strains isolated from food animal and human sources. The three fully sequenced IncA/C plasmids in the study shared sequence similarities with a number of other plasmids that were previously sequenced, however they had unique characteristics. There are more than 20 IncA/C plasmids that have been fully sequenced and archived in GenBank as of 8/15/2012, of these only two (JF503991 and JF714412) grouped together with near identical similarity. Thus these multidrug resistance plasmids, which share some common backbone sequences, are genetically diverse. Interestingly, pSH163_135 and pSH696_135 were closely related to each other; but they were distinct from many of the other IncA/C plasmids, such that portions of the transfer-associated regions, Tra 1 and 2, were missing compared to other IncA/C plasmids using Mauve. Some of the genes that were not present included *traNUW*, *ssb, bet* and multiple genes encoding hypothetical proteins [28]. These findings were consistent with previous results that showed multiple *Salmonella* isolates, including a *S.* Heidelberg isolated from a turkey, appeared to lack the portions of the transfer region in their IncA/C plasmids [19]. In addition, the Tn21 regions of pSH163_135 and pSH696_135, containing *bla*_TEM_, *aadB* and *cmlA,* were unique compared to other IncA/C plasmids [19], [29], but were similar to mobile elements in overlapping regions of plasmids from two *Klebsiella pneumoniae* plasmids (AY123253 and AJ704863).

The IncA/C plasmid pSH111_166 clustered more closely with multiple plasmids and contained the full concert of transfer genes, however there appears to be only a partial class 1 integron in the Tn21 element. The *int1* integrase gene was present, however other genes typical of the class 1 integron were absent including *qacEΔ* and *sul1*. pSH111_166 had a single copy of the *bla*_CMY-2_ gene, while pSH163_135 and pSH696_135 both had two copies. This variability in the *bla*_CMY-2_ region has been described previously [19].

Plasmids pSH163_120 and pSH696_117 were found to be similar to the IncFIB plasmids in multiple extraintestinal pathogenic *E. coli* isolates. The differences in plasmid sizes between isolates 163 and 696 were due to an approximately 3 kb section of plasmid sequence encoding a hypothetical protein in 163. The aerobactin and Sit iron transport operons encoded on these IncFIB plamids may play a role in the ability of *S.* Heidelberg to survive in the extraintestinal environments within the host where iron is in limited supply. The importance of these iron acquisition plasmids for extraintestinal survival in poultry has been demonstrated in *S.* Kentucky and APEC [30]–[32]. However, the exact role that these plasmids play in *S.* Heidelberg pathogenicity is currently unknown. It is probable that they may aid *Salmonella* serovars in colonization of poultry, since *S.* Heidelberg and Kentucky are the most commonly detected serovars in poultry and work in *S.* Kentucky has demonstrated their importance for colonization and extraintestinal disease in chickens [31]. The IncFIB plasmids of *S.* Kentucky and APEC contain additional virulence factors, including *iss* and other iron acquisition genes that are not present in pSH163_120 and pSH696_117 [31], [33]. The IncFIB plasmids in the current study also have host addiction genes (*vagCD* and *ccdAB*). These additional systems likely play a role in the stability of the plasmid in these isolates [34]–[38]. Indeed, multiple previous attempts to cure these plasmids from the strains have been unsuccessful (data not shown). In addition to the virulence genes, these plasmids also contain multiple antimicrobial resistance genes, thus, there is the potential for co-selection of increased antimicrobial resistance along with virulence. This phenomenon is potentially quite worrisome because the resistance and virulence genes are both bracketed by mobile genetic elements, including insertion sequences and transposons, and the plasmids themselves have been shown to be conjugative [16]. The potential role of these IncFIB plasmids in pathogenicity of human *Salmonella* infections has not been extensively studied and warrants potential evaluation.

The plasmid pSH1148_107 showed similarity to a *S*. Typhimurium IncI1 plasmid (CP002489) and contained a full concert of genes encoding conjugative transfer proteins. The main difference between pSH1148_107 and CP002489 is an insertion sequence within a Tn21 transposable element present in pSH1148_107 that contained genes associated with a class 1 integron, including *sul1*, *aadA, aacC* and *qacE*Δ, and heat shock proteins, *groES* and *groE*L. Overall, Tn21 element of pSH1148_107 showed greater similarity to the Tn21 element detected in an IncA/C plasmid (FJ621586) of *E. coli*, isolated from a dairy cow [39], which may indicate that this Tn21 element may be integrated into the IncI1 plasmid by transposition due to antimicrobial or other selection pressure. The IncI1 plasmids tend to be fairly narrow host range plasmids [40]; such that all previously sequenced plasmids with at least 50% query coverage with pSH1148_107 were isolated from either *E. coli* or *Salmonella.* The IncI1 plasmids have gained some interest due to their ability to acquire extended spectrum cephalosporin resistance [40]. While pSH1148_107 did not encode the cephalosporin resistance genes, it harbored resistance genes for Gen, Str and Sul associated with a class 1 integron. The insertion site for the integron was located in the same region as integrons from porcine *E. coli* plasmids pND11_107 (HQ114281), pWD4_103 (HQ114284) and pUMNF18_69 (CP002891) [40], suggesting that this site may be a potential hotspot for the incorporation of resistance genes into the IncI1 plasmids.

Interestingly, four of the five isolates in this study harbored two plasmids encoding resistance genes. In each case, there were resistance genes on the separate plasmids that encoded resistance to the same antimicrobials, indicating a duplication of resistance genes. There are a number of potential reasons why there may be multiple resistance plasmids in a strain. These could include the potential for conjugal co-transfer of the IncA/C plasmids, which lack all the needed transfer genes for self transfer, with those containing the needed transfer genes during periods of antimicrobial selective pressure. Additionally, many IncA/C plasmids have *bla*_CMY-2_ inserted into one of their *tra* genes, which could inhibit the transmissibility [19], [28], [41]. However, plasmids pSH163_135 and pSH696_135, which lacked multiple transfer-related genes, were able to be conjugally transferred [16]. This phenomenon is likely due to the presence of these additional non-IncA/C plasmids in these strains, including the IncFIB plasmids that contain a full complement of transfer genes or the smaller VirB/D4 T4SS plasmid that could possibly encode the conjugation machinery needed for the transfer (data not shown). Alternatively, there could be a potential fitness benefit to maintaining both the IncA/C and other plasmids in the strain. Johnson et al (2011) found a significant association between the detection of the IncA/C replication gene and IncHI2 replicons in *E. coli* isolated from porcine sources [40].

In addition to the antimicrobial resistance genes present, many of the plasmids contained disinfectant and metal resistance genes. The genes associated with quaternary ammonia compound resistance (*qacEΔ* and *sugE*) were co-located with antimicrobial resistance genes, either as part of class 1 integrons or with *bla*_CMY-2_ genes, respectively (Figure 1). Plasmid SH111_p227 contained several gene clusters associated resistance to metals, including cadmium, copper, cobalt, silver, tellurite and zinc (Figure 3). The disinfectant gene clusters were similar to those detected in the plasmid pAPEC-O1-R (DQ517526), a plasmid which can be conjugally transferred and encodes resistance to a number of compounds including benzylkonium chloride, copper sulfate, potassium tellurite and silver nitrate [27]. A difference between pSH111_227 and pAPEC-O1-R was the composition of antimicrobial resistance gene cluster that is located between the tellurite resistance and silver resistance gene clusters. In pAPEC-O1-R, the antimicrobial resistance genes included *aadA, aac3-VI* and *sul1*, whereas pSH111_227 harbored *strA, strB, tetA, tetC* and *tetD.* The IncA/C plasmid in isolates 111, 146, 163 and 696 also contained the mercury resistance operons, which in the fully sequenced plasmids are located as part of the transposons.

The plasmids sequenced in this study demonstrated some similarities to previously sequenced plasmids isolated from enteric organisms, however in each case there were unique regions contributing to plasmid diversity. Such diversity could potentially lead to favorable benefits to the bacteria occupying different ecological niches. Our ongoing work is focusing on the role of these plasmids in *Salmonella* virulence and determining what factors influence the dissemination of plasmids among enteric bacteria. Studies to understand the evolution and dissemination of plasmids are important to limit the future spread of increased virulence and antimicrobial resistance in *Salmonella* and other enteric pathogens.
